# Modifying social anxiety related to a real-life stressor using online Cognitive Bias Modification for interpretation^☆^

**DOI:** 10.1016/j.brat.2013.10.008

**Published:** 2014-01

**Authors:** Laura Hoppitt, Josephine L. Illingworth, Colin MacLeod, Adam Hampshire, Barnaby D. Dunn, Bundy Mackintosh

**Affiliations:** a Medical Research Council Cognition and Brain Sciences Unit, Cambridge, UK; b Norwich Medical School, University of East Anglia, UK; c Department of Psychology, University of Western Australia, Australia; d The Division of Brain Sciences, Imperial College London, UK; e Mood Disorders Centre, University of Exeter, UK

**Keywords:** Anxiety, Information processing, Interpretation bias, Attention bias, Cognitive Bias Modification

## Abstract

Modifying threat related biases in attention and interpretation has been shown to successfully reduce global symptoms of anxiety in high anxious and clinically anxious samples (termed Cognitive Bias Modification, CBM). However, the possibility that CBM can be used as a way to prevent anxiety associated with an upcoming real-life stressful event in vulnerable populations has yet to be systematically examined. The present study aimed to assess whether a two-week course of online CBM for interpretations (CBM-I) could reduce social evaluative fear when starting university. Sixty-nine students anxious about starting university completed five sessions of online CBM in the two weeks prior to starting university, or completed a placebo control intervention. Results indicated that CBM-I reduced social evaluative fear from baseline to day one of starting university to a greater extent than the placebo control intervention. Also, there was a greater reduction in state anxiety and a trend indicating a greater reduction in social evaluative fear in the CBM-I group at 4 weeks follow-up. Results suggest that CBM-I could be used as a preventative tool to help reduce anxiety specific to challenging life events.

## Highlights

• Cognitive Bias Modification (CBM-I) was tested as a preventative tool for real-life stress.
• Participants were students who reported anxiety about starting university.
• Participants were allocated to 2 weeks of CBM-I or placebo control before term began.
• Social evaluative fear was reduced in the CBM-I group but not in the placebo group.
• There was evidence of therapeutic effect up to four weeks post-intervention.

Threat-related cognitive biases in selective attention and in the interpretation of emotional ambiguity are known to be associated with high levels of anxiety. A recently developed technique, known as Cognitive Bias Modification, attempts to directly erode such biases in high and clinically anxious individuals in order to reduce anxiety symptoms. This is achieved using computer based tasks in which participants are required to repeatedly practice making positive interpretations of emotionally ambiguous words or scenarios (Cognitive Bias Modification for interpretation, CBM-I) or to decrease selective attention to threat-related stimuli (Cognitive Bias Modification for attention, CBM-A) (see Hertel and Mathews (2011) for a review).

So far, the majority of studies with high or clinically anxious volunteers have been conducted using CBM-A, with very encouraging results. For example, Amir, Beard, Burns, and Bomyea (2009) showed that eight 20 min sessions of CBM-A can significantly reduce anxiety in individuals with Generalized Anxiety Disorder. Amir, Bread, Taylor, et al. (2009) showed that eight sessions of CBM-A resulted in 50% of a social phobia sample no longer meeting diagnostic criteria following the intervention (as compared to 14% in the control condition), and therapeutic effects were maintained at a four month follow-up. Schmidt, Richey, Buckner, and Timpano (2009) found similar results when treating Generalized Social Anxiety Disorder. Eight sessions of CBM-A resulted in significant reductions in social and trait anxiety, and 72% of patients who received CBM-A no longer met DSM-IV diagnosis criteria (as compared to 11% in the control group). Again therapeutic benefits were maintained at 4 month follow-up.

Recent studies suggest that CBM-I also may be effective in reducing elevated anxiety. For example, Mathews, Ridgway, Cook, and Yiend (2007) found that CBM-I served to lower trait anxiety in a high anxious population over four sessions. Similarly, Salemink, van den Hout, and Kindt (2009) also demonstrated that multiple CBM-I sessions led to significant reductions in trait anxiety, as well as state anxiety and general psychopathology. Beard and Amir (2008) found reductions in social anxiety symptoms following CBM-I in a highly socially anxious population. Hirsch, Hayes, and Mathews (2009) showed that CBM-I significantly reduced levels of worry in a sample of high worriers. We are aware of only four studies that have attempted to use CBM-I in a clinically anxious population where clinical status of the participants was confirmed using diagnostic interviewing. Two of these used a combined package of CBM-I and CBM-A, and showed significant reductions in state and trait anxiety in those with Generalized Anxiety Disorder or Social Anxiety Disorder (Brosan, Hoppitt, Shelfer, Sillence, & Mackintosh, 2011), and in symptoms of social anxiety and a behavioural indicator of performance anxiety in those with Social Anxiety Disorder (Beard, Weisberg, & Amir, 2011). Amir and Taylor (2012) used CBM-I to treat Social Anxiety Disorder, and showed CBM-I could significantly reduce clinician rated social anxiety, trait anxiety and depression. The remaining study found that CBM-I reduced negative thought intrusions in individuals with Generalized Anxiety Disorder (Hayes, Hirsch, Krebs, & Mathews, 2010).

Of particular interest to clinicians and researchers is whether these techniques could be used as a preventative strategy in populations vulnerable to the future development of anxiety disorders, to reduce anxiety and distress associated with the anticipation of an upcoming challenging life event, and/or the anxiety associated with being in the middle of challenging circumstances. Consistent with this possibility, there is encouraging laboratory evidence that CBM-I can reduce reactions to subsequent stressful events in non-anxious populations. For example, Wilson, MacLeod, Mathews, and Rutherford (2006) first demonstrated that modifying interpretive bias in either a threat or non-threat direction impacted on anxiety vulnerability in response to watching a film containing scenes of real-life accidents. Participants in the threat interpretation training condition showed greater increases in anxiety whilst watching the film as compared to participants in the non-threat interpretation training condition. Using an identical stressor, Mackintosh, Mathews, Yiend, Ridgeway, and Cook (2006) demonstrated that such effects of CBM-I on anxiety vulnerability can last for at least 24 h post-training.

The critical issue from a clinical perspective is of course whether these effects hold when examining more ecologically valid stressors. To our knowledge, only one published study has attempted to utilize CBM to ameliorate negative emotional response to a real life stressful event, and this study evaluated the impact of CBM-A rather than CBM-I. See, MacLeod, and Bridle (2009) investigated whether CBM-A could reduce the distress associated with a move to Australia and starting university, in a non-clinical sample of Singaporean students. Two and a half weeks before they made the move, participants completed pre-intervention measures of state and trait anxiety. Across the ensuing 15 days the students either completed daily online CBM-A sessions (CBM-A condition) or else completed a placebo version of the task which was not configured to modify attentional bias (no-training control condition). On arrival in Australia the students were asked to fill out post-intervention measures of state and trait anxiety. The results indicated that CBM-A, compared to the control condition, was successful in reducing threat-related attentional bias, and this resulted in significantly lower levels of trait anxiety, and attenuated state anxiety responses to the event of moving to Australia. Unfortunately, in this study there was no longer term follow-up to determine if the benefits of CBM persisted beyond the first day. Moreover, this study did not select participants who were particularly vulnerable to anxiety concerning the potential stressor, and it is important to establish if the effects will hold in these individuals. Nevertheless, this initial study paved the way for further work investigating the use of CBM in a preventative way, to reduce threat-related bias before a challenging real-life event.

The aim of the present study was to assess for the first time whether similar findings could be attained using CBM-I (designed to modify selective interpretation) in relation to anxiety in participants experiencing a similar stressful life event and in a sample vulnerable to anxiety. Furthermore, we aimed to assess whether such beneficial effects last beyond the initial day of the stressful event, to help individuals continue to cope better emotionally with the event. Finally, we were also interested in examining whether training one type of bias (interpretive bias) would lead to transfer to another type of bias (attentional bias). Such transfer effects of training have received little attention in the literature and we know of only one published study that has assessed transfer of interpretive training to attentional bias. Amir, Bomyea, and Beard (2010) modified interpretive bias using a Word Sentence Association Paradigm and examined its impact upon attentional bias (as assessed by a modified Posner task). They found that interpretive training successfully modified both interpretive and attentional biases. Such findings are important theoretically in that they support models of anxiety which propose that information processing biases work together and stem from a common mechanism to maintain symptoms (e.g., Bishop, 2007; Mathews & Mackintosh, 1998). Furthermore, therapeutically, if training one bias can impact upon another bias it tentatively implies that focusing an intervention on the modification of one cognitive bias (e.g., interpretation), as opposed to concurrently training other biases (e.g., attention) could be sufficient.

In the present study, the challenging event was the move to university (University of East Anglia in the UK) for students who had self-identified as being anxious about this upcoming event. This is a stressor that is likely to lead to significant elevations in anxiety in the weeks prior to start of university due to anticipatory processing, and for anxiety to continue to be elevated once university actually starts. We aimed to attempt to use CBM-I to increase positive interpretive bias in the weeks leading up to this potential stressor, such that those in the intervention group would be more able to interpret the event in a positive way and situations that they might find themselves in a more positive manner than those in the control group, thereby reducing anticipatory anxiety.

The students were offered five sessions of CBM-I, delivered online in their home setting, across the two weeks prior to the start of the university semester. Pre and post-intervention, measures were taken not only of interpretive bias, but also of attentional bias, to determine transfer of training from interpretation to attentional bias (as previously found by Amir et al., 2010; but not yet replicated). We also assessed social anxiety, and more general anxiety, prior to the CBM-I intervention, on the day of commencing university, and at a follow-up four weeks into the university semester to examine longer terms effects of CBM-I. This allowed us to assess not only anxiety associated with the actual commencement of university (day one) but also anxiety levels during the first semester of university (four-week follow-up), which would give an indication of how participants are coping emotionally with the continuing stress associated with this new situation. The CBM-I training focused on modifying the interpretation of emotionally ambiguous social scenarios, related to the kind of anxieties thought likely to be associated with beginning university; social evaluative situations (such as whether one will be liked by one's new friends) and performance evaluative situations (such as how well one will be able to get on with one's studies). We therefore had as our main outcome variable Fear of Negative Evaluations (FNE, Watson & Friend, 1969), with the prediction that CBM-I should significantly reduce this fear, with effects being maintained at follow-up, as compared to the placebo control condition. We also predicted that CBM-I would reduce state anxiety, and might even reduce trait anxiety (assessed using the Spielberger State Trait Anxiety Inventory; STAI, Spielberger, Gorsuch, Lushene, Vagg, & Jacobs, 1983).

## Method

### Design overview

Prospective university students who were anxious about starting their course were recruited over the summer before university began, and were randomly assigned to receive either the online CBM-I intervention (5 sessions over 2 weeks) or the online placebo control intervention (matched for number of sessions) across the two weeks before the semester began. Pre and post intervention measures of interpretive and attentional bias were taken. Fear of negative evaluation, state anxiety, trait anxiety, together with positive and negative affect, were assessed pre-intervention, on day one of the life stressor (day one of university) and four weeks into the semester (four week follow-up).

### Participants

All prospective UK students starting at the University of East Anglia in the academic year 2010–2011 were invited to take part in the study via email during prior summer. Overseas students were not included in the recruitment drive in order to ensure additional stresses of moving to a new country would not affect the results. Inclusion criteria were that they were between 18 and 35 years old, reported having no current mental health issue and were new students about to begin university on an undergraduate course for the first time at UEA with anxiety about starting university. The first 100 students to respond (and who met these criteria) were given further information about the study, and of these sixty-nine (55 female) consented to take part. Six further participants are not reported in the analysis due to withdrawing from the study (*n* = 5) or not completing a minimum of at least 4 out of 5 training sessions (*n* = 1). Participants were randomly allocated to either the CBM-I intervention or placebo control intervention, with the restriction that sample size was kept approximately equal between these conditions (CBM-I *n* = 35, placebo control *n* = 34).

### Materials and measures

#### Cognitive Bias Modification for interpretation materials (CBM-I)

CBM-I was delivered online with participants logging on to a website with a password. The CBM-I was based on that originally used by Mathews and Mackintosh (2000), with the addition of a brief rationale for taking part in the task. In this rationale participants were told that imagining themselves in a range of fictitious scenarios may help to reduce feelings of anxiety in real life, because by using their imagination it helps to get used to being in various different situations. In each of the 5 CBM-I sessions participants were presented with two blocks of 13 vignettes to read (and imagine themselves in), and each block and session contained new sets of vignettes. Each vignette was presented a line at a time, self-paced, and described an emotionally ambiguous situation relevant to student concerns. The final word of the scenario description, which was presented with some letters missing, always resolved the ambiguity in a non-threatening way. For example:

*You have not spent as much time with your new flatmates as you thought you would. Some of them are quite different from you. You decide to ask your flatmates if they fancy having a party, and their response shows they think your idea is b-illi-nt (target solution: brilliant)*

As they were reading each scenario, participants were asked to imagine themselves in the situation, and were told to use their image to work out what the missing letters of the final word were. Following each scenario a comprehension question was presented which, when answered correctly, confirmed the positive interpretation of the scenario. Participants were given feedback on whether they answered this question correctly.

#### Placebo control materials

In a similar way to the CBM-I condition, the placebo intervention was delivered online with participants logging on to a website with a password. Participants completed 5 sessions of “brain training” tasks (provided by Cambridge Brain Sciences). These tasks trained general visuospatial ability, rather than interpretive bias, but they required the control participants to spend an approximately equal time at the computer as participants in the CBM-I condition (although as the CBM-I intervention is self-paced it was impossible to control exactly for time taken). Control participants were given a rationale that was piloted to ensure a similar expectation (to participants in the CBM-I group) that this should be an effective intervention. In the rationale participants were told that these tasks should train their executive abilities, which should help them shift their focus from their worries and therefore find it easier to begin university. Importantly, these tasks used neutral rather than emotional material to ensure that the control condition did not also reduce anxiety. There is evidence that brain training only results in changes in affective information processing if emotional (and not neutral) material is processed (Schweizer, Hampshire, & Dalgleish, 2011).

#### Assessment of interpretive bias (recognition memory task)

The recognition task was made accessible online to participants via the same website as their intervention. It was presented immediately pre- and post-intervention, together with the attentional bias assessment task (see below). The recognition task was based on the version originally used by Mathews and Mackintosh (2000). In this interpretive bias assessment test participants were first asked to read, and imagine themselves in, a series of ten emotionally ambiguous scenarios, such as:

*You participate in a health and safety refresher course organised by the university. The instructor asks a question and no one in the group volunteers an answer, so he looks directly at you. You offer a reply, thinking about how your answer must be sounding to the others.*

After each scenario participants were asked to rate how vivid their image was and to answer a comprehension question to ensure they read the material thoroughly. As the recognition test was to be repeated pre and post-intervention, participants were told from the start that these scenarios would be followed by a memory test. In the recognition memory phase, participants were presented with four sentences for each of the scenarios they had previously read. Two of these sentences were targets, in that they represented possible interpretations of the scenario, one of which was negative and one positive. The other two sentences were “foils”, in that they were not possible interpretations of the scenario, though again one was negative and one positive. For each sentence participants were asked to rate from 1 to 4 how similar it was to the scenario that they previously read (1 = not similar at all to 4 = very similar). If participants were displaying a positive interpretive bias we would expect them to rate the positive targets as being most similar to the previous emotionally ambiguous scenarios as compared to the negative targets. For the previous given example scenario the recognition sentences were:

*You answer the question, thinking that the others may be quite impressed (positive target)*

*You answer the question, thinking how ignorant you may seem (negative target)*

*You answer the question, pleased that you have such an interesting teacher (positive foil)*

*You answer the question, realising you are irritated by this teaching style (negative foil)*

To ensure that participants understood the task, both pre- and post-intervention they took part in a practice version of this test with neutral material. This practice consisted of four neutral scenarios in the encoding phase followed by a recognition memory test of these scenarios. To enable pre and post-intervention testing two lists of ten emotionally ambiguous scenarios were developed, and order of presentation was counterbalanced across participants.

This version of the recognition task has been widely used to assess change in interpretive bias due to Cognitive Bias Modification procedures and has well established validity (e.g., Mackintosh et al., 2006; Mathews & Mackintosh, 2000; Salemink, van den Hout, & Kindt, 2007; Yiend, Mackintosh, & Mathews, 2005). Recently, Salemink and van den Hout (2010) demonstrated that this version of the recognition test successfully differentiates between participants scoring high and low on neuroticism, and that the test is not sensitive to changes in mood state. This latter finding is particularly important for CBM research as it suggests that changes on this measure following CBM do in fact reflect changes in cognitive bias rather than fluctuations in mood state that may result from task participation.

#### Assessment of attentional bias (dot-probe task)

The dot-probe task was made accessible online to participants via the same website as their intervention. It was presented immediately pre- and post-intervention in the same assessment session as the interpretive bias test (see above). On each trial in this task participants were first required to focus initial attention on a central fixation point on a computer screen. After 500 ms, the fixation disappeared and was replaced by two words, one appearing just above and the other just below fixation. One word was always social threat related (e.g. *failure*) while the other was always non-threatening (e.g. *feature*). After 500 ms these words both disappeared and in the screen position where either one of the words had been shown a probe arrow appeared, pointing either left or right (< or >). Participants were instructed to identify whether the probe was pointing left or right, and respond by pressing a corresponding key. After an inter-trial interval of one second the next trial began. In this task, an attentional bias towards negative stimuli is revealed by a speeding to discriminate the identity of those probes that appear in the locus where threat words were just presented, relative to probes that instead appear in the locus where non-threat words were just presented. Two sets of 36 word pairs were constructed, with the members of each pair being matched on word frequency and number of letters, but differing in emotional tone. This allowed the pre and post-intervention test of attentional bias to be delivered using different stimulus materials. These two word pair sets were matched in terms of rated threat value of the threat words and the assignment of each set to the two assessment sessions was counterbalanced across participants. Threat words appeared above and below fixation an equal number of times, the probe was presented in the prior location of the threat word and the non-threat word an equal number of times, and this probe pointed to the left or right an equal number times (resulting in 288 trials per assessment, split into four blocks of 72).

#### Emotional measures

Participants were asked to complete a series of questionnaires pre-intervention, on the first day of the life stressor (the first day when starting at UEA), and at follow-up (four weeks after starting at UEA). The Fear of Negative Evaluation scale (FNE; Watson & Friend, 1969), was the main assessment instrument, which measures changes in symptoms relating to fear of evaluation in social situations. This relates directly to the focus of the CBM-I intervention, which aims to modify changes in appraisal in the context of a social stressor. The FNE is a 30-item true/false questionnaire which refers to expectation and distress related to negative evaluation from others in social situations in the present. The scale shows good test–retest reliability over a one week period (*r* = 0.94) and internal consistency (*α* = 0.88–0.94).

The State Trait Anxiety Inventory (STAI; Spielberger et al., 1983) was used to assess level of state anxiety at the specific time when the inventory was completed and general trait anxiety. This is a broader measure of anxiety and need not involve social or evaluative concerns. The state and trait and state sections of the inventory each have 20 items which are rated on a four point scale. The STAI has demonstrated good test–retest reliability, with alpha coefficients reported by Spielberger et al. (1983) ranging from around 0.89 to 0.92.

To assess any effect on levels of positive or negative affect we used the Positive and Negative Affect Schedule (PANAS; Watson, Clark, & Tellegen, 1988). It is a 20 item self report scale in which participants are presented with words related to feelings associated with positive or negative affect, and must rate on a scale of 1–5 the extent to which they feel that way in general (1 = very slightly or not at all, 5 = extremely). The PANAS shows good test–retest reliability (coefficients: Positive Affect = .68; Negative Affect = .71) and internal consistency (alpha coefficients: Positive Affect = .87; Negative Affect = .88).

### Procedure

Participants were emailed the information sheet and consent form, and if happy to continue signed the consent form and posted it back to the researcher. Prior to the study, participants were posted a pack containing the first two sets of questionnaires. On day one of the intervention, scheduled two weeks prior to the students' first day at university, participants were asked to fill out the first set of questionnaires. All participants then completed the attention bias assessment, followed by the interpretive bias assessment online. This was followed by the first CBM-I or placebo control session. Over the next two weeks participants completed three further sessions of CBM-I or placebo control intervention, spaced across the period so as to avoid completing sessions on consecutive days. On the final day, which was the day before participants started university, they completed the final CBM-I or placebo control intervention session, followed by the post-intervention test of attentional and interpretive bias, in that order. Two different material sets were developed for both the attentional and interpretative bias tests, one of which was used pre-intervention and one of which was used post-intervention. Which version of the materials participants viewed pre- and post-intervention was counterbalanced across participants in each group to eliminate materials effects. On arrival at UEA, on their first day of university, participants were asked to complete the second set of the questionnaires and put these completed forms into a secure drop-box. Four weeks into the university semester participants visited the laboratory for the follow-up session. In this session participants were asked to complete the final FNE, STAI and the PANAS questionnaires, received their payment for taking part (approximately £6 per hour), and were fully debriefed. Participants in the placebo control condition were offered access to the online CBM-I materials.

## Results

### Participant characteristics

At baseline (pre-intervention) there was no significant difference between groups in terms of Fear of Negative Evaluations (FNE), *F*(1, 66) = 2.65, *p* = .11, Spielberger State Trait Anxiety Inventory (STAI), state, *F*(1, 66) = 2.73, *p* = .10; trait, *F*(1, 67) = 2.80, *p* = .10, either subscale of the Positive and Negative Affect Schedule (PANAS), positive affect, *F* < 1; negative affect, *F*(1, 66) = 1.95, *p* = .17 (see Table 1). Groups also did not differ in baseline interpretive bias, *F* < 1, or attentional bias, *F* < 1.

### Impact of CBM-I on interpretive bias

A mixed model ANOVA was run on the recognition test similarity ratings, with time (pre–post intervention), statement type (target, foil) and statement valence (positive, negative) as the within subjects factors and training group (CBM-I or placebo control) as the between subjects factor, (see Table 2). There were main effects of time, *F*(1, 51) = 14.45, *p* < .001, *η*^2^ = 0.22; statement type, *F*(1, 51) = 335.74, *p* < .001, *η*^2^ = 0.87; and statement valence, *F*(1, 51) = 14.83, *p* < .001, *η*^2^ = 0.23, and an interaction between time and statement valence, *F*(1, 51) = 33.82, *p* < .001, *η*^2^ = 0.40. Similarity ratings of positive statements increased over the course of training, as compared to ratings of negative statements (indicating a more positive bias in both groups post-intervention as compared to pre-intervention). Critically, these effects were qualified by a significant 3-way interaction between time, valence and group, *F*(1, 51) = 4.80, *p* = .03, *η*^2^ = 0.09, which suggests that the degree of improvement in positive bias depended on intervention group. There was no further interaction involving type (target vs. foil), *F* < 1, suggesting that the effect generalized to both targets and foils. To investigate the three way interaction further, a bias index was computed by subtracting similarity ratings for negative statements from similarity ratings for positive statements, collapsed across targets and foils. Paired sample *t*-tests suggested that this bias became significantly more positive in both groups, CBM-I group (pre *M* = −0.08, *SD* = 0.43; post *M* = 0.52, *SD* = 0.44), *t*(26) = −5.14, *p* < .001, placebo control group (pre *M* = 0.03, *SD* = 0.40; post *M* = 0.30, *SD* = 0.54), *t*(25) = −2.59, *p* = .007. However, the nature of the three-way interaction was that this tendency for the bias to become more positive was greater for the CBM-I group than for the control group, and consistent with this participants in the CBM-I group showed a significantly more positive interpretive bias post-intervention as compared to participants in the placebo control group, *t*(60) = 2.17, *p* = .03.

### Impact of CBM-I on attentional bias

In line with See et al. (2009), to reduce the influence of outliers probe discrimination latencies less than 200 ms or greater than 2000 ms were eliminated and median latencies were computed for each condition in the probe task. These latencies were subject to a mixed model ANOVA in which time (pre or post training) and probe location (probe in locus of threat or non-threat word) were within subjects factors, and group (CBM-I or placebo control) was a between subjects factor. There was a main effect of time, indicating an overall speeding of probe discrimination latencies (pre *M* = 476.65, *SD* = 57.78; post *M* = 456.55, *SD* = 56.39), *F*(1, 62) = 17.21, *p* < .001, *η*^2^ = 0.22, and an interaction between time and group, indicating that this speeding was relatively greater in the placebo control group, *F*(1, 62) = 5.53, *p* = .02, *η*^2^ = 0.08, (pre–post mean difference CBM-I = 8.99, *SD* = 42.95; placebo = 32.52, *SD* = 36.22). However, there was no evidence of a three-way interaction between time, probe position valence and group, *F* < 1. This suggests that CBM-I did not modify selective attentional response to threatening information.

### Impact of CBM-I on anxiety and mood pre-training to post-training (day one of stressor)

In terms of our main dependent variable, which was Fear of Negative Evaluations, we ran a mixed model ANOVA with assessment point as the within subjects variable (pre and post training) and group as the between subjects variable (CBM-I or placebo control). There was no significant main effect of assessment point, *F*(1, 66) = 1.46, *p* = .23, *η*^2^ = 0.022, or group, *F* < 1, but critically there was a significant interaction between assessment point and group, *F*(1, 66) = 5.72, *p* = .02, *η*^2^ = 0.08. Planned comparisons suggested that there was a significant decrease in social evaluative fear in the CBM-I group, *t*(33) = 2.33, *p* = .03, as compared to no significant change in the placebo control group, *t* < 1 (see Table 1).

The effect of the intervention on trait and state anxiety were analysed in the same way. There were no significant main effects of assessment point, state: *F* < 1, trait: *F* < 1, or group, state: *F*(1, 66) = 1.02, *p* = .32, *η*^2^ = 0.02, trait: *F*(1, 65) = 2.15, *p* = .15, *η*^2^ = 0.03. There were no significant assessment point by group interactions, state: *F*(1, 66) = 1.34, *p* = .25, *η*^2^ = 0.02, trait: *F* < 1. Similarly, in terms of positive and negative affect as measured by the PANAS, there were no significant main effects of assessment point, positive affect: *F* < 1, negative affect: *F* < 1, or group, positive affect: *F*(1, 62) = 1.72, *p* = .20, *η*^2^ = 0.03, negative affect: *F*(1, 62) = 2.34, *p* = .13, *η*^2^ = 0.04. There were no significant assessment point by group interactions, *F* values < 1 (see Table 1).

### Impact of CBM-I on anxiety and mood pre-training to 4-week follow-up

Comparing fear of negative evaluation from pre-training to follow-up there was a significant main effect of assessment point, *F*(1, 57) = 25.33, *p* < .001, *η*^2^ = 0.31, suggesting an overall decrease in social evaluative fear. There was no main effect of group, *F* < 1, but there was a near-significant interaction between assessment point and group, *F*(1, 57) = 3.29, *p* = .08, *η*^2^ = 0.06. Planned comparisons suggested decreases in social evaluative fear in both the CBM-I group, *t*(28) = 4.25, *p* < .0001, and the placebo control group, *t*(29) = 2.68, *p* < .01, although the near-significant interaction suggests that the size of the decrease in the CBM-I group was greater than that of the placebo control group (see Table 1).

In terms of state anxiety, again there was a main effect of assessment point, suggesting an overall decrease in state anxiety, *F*(1, 57) = 23.38, *p* < .001, *η*^2^ = 0.29. There was no main effect of group, *F* < 1, but there was a significant interaction between assessment point and group, *F*(1, 57) = 4.53, *p* = .04, *η*^2^ = 0.074. Planned comparisons suggested a significant decrease in state anxiety in the CBM-I group, *t*(28) = 5.09, *p* < .001, and a near-significant decrease in the placebo control group, *t*(29) = 1.86, *p* = .07, with the significant interaction suggesting that the size of the decrease in the CBM-I group was greater than that of the placebo control group (see Table 2). For trait anxiety, there was again a significant main effect of assessment point, which indicated an overall decrease in trait anxiety, *F*(1, 57) = 13.18, *p* = .001, *η*^2^ = 0.19, but there was no main effect of group, *F*(1, 57) = 1.87, *p* = .18, *η*^2^ = 0.03, and no assessment point by group interaction, *F* < 1 (see Table 1).

In terms of positive and negative affect, there was no main effect of assessment point on positive affect, *F*(1, 55) = 1.61, *p* = .21, *η*^2^ = 0.03, but a main effect of assessment point on negative affect (suggesting an overall reduction in negative affect), *F*(1, 56) = 46.18, *p* = .001, *η*^2^ = 0.45. There were no significant main effects of group for either positive affect, *F* < 1, or negative affect, *F* < 1, and no significant assessment point by group interactions for either positive affect, *F* < 1, or negative affect, *F*(1, 56) = 1.21, *p* = .28, *η*^2^ = 0.021 (see Table 1).

## Discussion

Our results suggest that CBM-I is effective in reducing social evaluative fear associated with the stressful life event of starting university. Participants in the CBM-I condition show significantly greater reductions as compared to the placebo control group in social evaluative fear from baseline to day one, and trend greater reductions from baseline to four-week follow-up. This suggests that CBM-I successfully modified social evaluative fear associated with the anticipation of encountering social performance situations at university and to some extent the ongoing fear of social and performance evaluation encountered during the semester. Our results also suggest that CBM-I can successfully reduce state anxiety associated with an ongoing potential life stressor, as the CBM-I group showed greater reductions in state anxiety from pre-training to follow-up as compared to the placebo control condition.

These results are consistent with previous research findings suggesting that CBM for attention (as opposed to interpretation, as in this study) might be effective in reducing anxiety associated with a real-life stressor, and that it can be delivered effectively online with no experimenter contact (See et al., 2009). However, importantly our results extend these findings. We found for the first time that CBM for interpretation can be used in a similar way, to reduce social fears associated with a challenging life event in an anxious group. Furthermore, we found that this effect can extend to at least four weeks following the end of the CBM-I intervention.

It is noteworthy that there appeared to be a specific effect of the intervention on social evaluative fear (and state anxiety at follow-up), rather than a more general impact on trait anxiety and positive and negative affect. It is likely that the specific effect on fear of negative evaluations is due to the training materials being confined to social evaluative situations. The finding that more general measures of trait anxiety and mood were not modified by CBM-I suggests that emotional concerns related to other domains may have been left unaffected by the intervention, perhaps due to the specific training materials used. The additional impact of CBM-I on state anxiety at follow-up might be due the reduction in fear in encountering social situations impacting on participants' moment to moment feeling of anxiety. Alternatively, it might be that the questionnaires differ in how malleable they are, as a function of their content. The PANAS and STAI trait scale items relate to how the participant feels “in general” rather than in the moment, and it seems likely that changing general trait measures is more difficult than changing measures of a specific fear. Given that trait anxiety is assessed by having participants rate how often they generally find themselves experiencing anxiety, it will require a much more extended period of change to state anxiety levels before this will result in changes to trait anxiety scores. Nevertheless, some previous CBM-I studies have been successful in reducing scores on such trait measures (e.g., Mathews et al., 2007; Salemink et al., 2009).

At face value it is surprising that state anxiety did not significantly increase from pre-intervention to the first day of university in the control group, and actually dropped in the CBM-I group. This could be interpreted as showing that the start of the university is not actually experienced as stressful by participants. We feel this is unlikely for two reasons. First, all participants were selected on the basis that they said they were worried about starting university. Second, and critically, much of anxiety is anticipatory in nature, It is entirely plausible that participants feel just as anxious in the build up to the stressor as when the stressor actually occurs, due to a range of anticipatory simulations of the social threat the event will pose. In this light, the drop in anxiety in the active group is likely to reflect alterations in this anticipatory processing.

It is also interesting that rather than modifying interpretive bias per se, the intervention appeared to have induced a more general positive bias on both targets and foils in the recognition test (the effects of training were no less evident on the emotional foil sentences than on the target sentences representing alternative possible interpretations of emotionally ambiguous scenarios). The present study is not alone in finding that this CBM-I procedure can change the subjective familiarity of emotional foils as well as targets in this recognition task. Previous studies have also found evidence of this general bias effect of the CBM-I training (e.g., Hoppitt, Mathews, Yiend, & Mackintosh, 2010; Turner, Hoppitt, Hodgekins, Mackintosh & Fowler, 2011). In some studies there has been evidence that CBM-I training induced both an interpretive bias and additionally a more general tendency to report foils of the trained valence as being more familiar (e.g., Hoppitt et al., 2010), but such a distinction was not clear in the present data.

It is also noteworthy that in the present study there was no evidence that training transferred from interpretive modification procedure to a test of attentional bias. This is not consistent with previous findings which have shown successful transfer from interpretive training to attentional bias (Amir et al., 2010) and does not support theoretical models arguing for a common information processing mechanism underpinning anxiety (Bishop, 2007; Mathews & Mackintosh, 1998). However, there were procedural differences in terms of both the training phase and test phase between the present study and Amir et al. (2010). Whereas the present study used interpretive training based on participants imagining themselves in emotionally ambiguous scenarios in which active generation of positive endings is encouraged, Amir et al. (2010) asked participants to make speeded relatedness judgments between words and sentences. In the Amir et al. (2010) study, participants are given “You are Correct!” feedback if they say that a word and sentence which create a positive meaning are related (e.g., funny: People laugh after something you said) and “You are Incorrect!” feedback if they say that a word and sentence which create a negative meaning are related (e.g., embarrassing: People laugh after something you said). Subsequently, in the test of attentional bias, we utilized a visual probe task, whereas Amir et al. (2010) used a modified Posner task. It could be argued that transfer might be easier between the two tasks employed by Amir et al. (2010) due to the fact that both training and test appear to reply on speeded judgments, and perhaps relatively more automatic processing (whereas in the present study, the training involves more elaborative processing of emotional ambiguous scenarios which might engage a more controlled level of processing).

It is also possible that our null attentional bias results could be due to the reliability of assessing attentional bias via a website. However, web-based assessment of cognitive processes now represent a well-established approach, and have been previously used successfully and reliably in the assessment of attentional bias. For example, MacLeod, Soong, Rutherford and Campbell (2007) and See et al. (2009) show that both anxiety linked individual differences in attentional bias and CBM induced change in attentional bias can be detected using web-delivery of the same visual-probe task used in the present work. A critical issue is ensuring the accuracy of reaction time data using online platforms. In the present study we use a previously validated web-based platform developed by Cambridge Sciences, which has relatively small RT error (20–50 ms), comparable to offline tasks programmed in other experimental software. For example, this has been used to assess the effectiveness of emotional brain training with an online version of the emotional Stroop as the main outcome measure (Schweizer et al., 2011).

The study is not without limitations. First, due to the self-paced nature of the CBM-I intervention, we could not control exactly for time taken by the two interventions and were unable to test this. However, the tasks were designed to take approximately the same time and we would not expect any substantial differences in this regard. Second, other than self-report data on fear of negative evaluations, anxiety and depression, we do not have data on the level of impairment that participants were experiencing in terms of their anxious apprehension specific to starting university. It is likely that some participants would meet diagnostic criteria for various anxiety disorders, whereas others would fall in subclinical ranges. It is possible that differences in the level of clinical impairment might have impacted upon the effectiveness of the intervention, although a recent meta-analysis has found no link between clinical severity and outcome of CBM (Hallion & Ruscio, 2011). Third, the fact that we administered the attentional and interpretative bias tests in a fixed order means that we cannot rule out order confounds in the data. However, we feel it is unlikely that completion of the attentional bias test will have affected scores on the subsequent interpretive bias test. In the attentional bias test, the number of trials in which the probe appeared in the position of the threat word was equal to the number of trials in which it appeared in the position of the non-threat word, so it should not have had an overall effect on processing of emotional information. Even if any effects on processing did occur, we would not expect them to vary across groups or from pre- to post-training, meaning that this would not substantially influence our findings. Fourth, we have no way of establishing that participants completed the pre-intervention assessment measures at the correct time as they were posted out to participants, but we have no reason to expect that this would systematically differ between the groups. Finally, while the recognition task we used to measure interpretative bias has well established validity, its reliability has yet to be formally examined. Further work is needed to fully establish the psychometric properties of this instrument.

These limitations not withstanding, the present study has some potential therapeutic implications. It is possible that CBM could be used as a preventative strategy to reduce anxiety and fear associated with an inevitable upcoming stressful event. The present study and that reported by See et al. (2009) assessed the capacity of CBM to modify fear and anxiety associated with a major life challenge, such as moving to a new area (or country) and university. However, the results of the present study suggest that when using CBM-I it might be important to tailor the training material to the stressor that is to be encountered. The present study was effective at reducing social evaluative fear (which was our major target), and state anxiety at follow-up, but did not modify more general trait anxiety or mood. It is important to acknowledge that while the CBM-I group showed greater reductions in anxiety than the control group, they did not significantly differ in absolute levels of anxiety at the post-intervention or follow-up assessments. Therefore, these clinical implications should be viewed as preliminary at the present time. Given the small number of studies that have attempted to use CBM-I in a preventative way and the fact that effects are not totally clear cut in these existing studies, this area would benefit from further research.

In conclusion, the present study extends previous research demonstrating that Cognitive Bias Modification can serve to alleviate anxiety symptoms. In contrast to previous studies, the present experiment tested whether interpretive bias modification could reduce social evaluative fear associated with an upcoming real-life stress in an anxious population. Results suggested that CBM-I was effective at alleviating social fear associated with the stress of starting university, and that its effects last for at least four weeks.

## Figures and Tables

**Table 1:** Means at pre-training (baseline), post-training (day one) and follow-up (four weeks post-training) for Fear of Negative Evaluations (FNE), State and Trait Anxiety (STAI-S and STAI-T) and positive and negative affect as assessed by the Positive and Negative Affect Schedule (PANAS-P and PANAS-N), split by condition (Cognitive Bias Modification for interpretation, CBM-I, or Placebo Control).

	CBM-I			Placebo control		
	Pre	Post	Follow-up	Pre	Post	Follow-up
FNE	21.73 (6.51)	18.91 (7.64)	16.23 (8.27)	19.09 (6.91)	19.88 (7.11)	17.07 (7.55)
STAI-S	45.32 (12.28)	43.74 (13.29)	35.33 (6.90)	41.00 (9.09)	44.18 (12.56)	37.43 (10.64)
STAI-T	47.52 (10.03)	44.63 (11.35)	42.30 (9.40)	43.44 (9.92)	43.18 (9.77)	39.37 (9.43)
PANAS-P	35.12 (5.15)	35.58 (7.00)	33.79 (5.01)	34.47 (3.96)	33.81 (4.33)	34.27 (4.02)
PANAS-N	22.47 (6.96)	21.48 (8.32)	17.03 (5.51)	20.38 (5.27)	19.72 (6.22)	16.87 (4.05)

*Note.* Standard deviations in parentheses.

**Table 2:** Means at pre-training and post-training for latencies (in milliseconds) to respond to probes appearing in the location of threat and non-threat words in the dot probe test of attentional bias, and for similarity ratings of the four sentence types used in the recognition test of interpretive bias, split by condition (Cognitive Bias Modification for interpretation, CBM-I, or Placebo Control).

		CBM-I		Placebo control	
		Pre	Post	Pre	Post
Dot probe test	Threat words	465.85 (58.72)	456.82 (63.69)	488.60 (54.69)	455.53 (42.65)
	Non-threat words	466.44 (58.95)	457.50 (70.87)	488.13 (59.73)	456.17 (43.41)

Recognition test	Negative targets	2.51 (0.52)	2.16 (0.50)	2.46 (0.60)	2.34 (0.55)
	Positive targets	2.23 (0.46)	2.63 (0.40)	2.21 (0.39)	2.51 (0.47)
	Negative foils	1.52 (0.40)	1.50 (0.35)	1.40 (0.29)	1.56 (0.33)
	Positive foils	1.62 (0.46)	2.05 (0.50)	1.58 (0.42)	1.87 (0.50)

*Note.* Standard deviations in parentheses.

## References

[bib1] Amir N., Beard C., Burns M., Bomyea J. (2009). Attention modification program in individuals with generalized anxiety disorder. Journal of Abnormal Psychology.

[bib2] Amir N., Beard C., Taylor L.T., Klumpp H., Elias J., Burns M. (2009). Attention training in individuals with generalized social phobia: a randomized controlled trial. Journal of Consulting and Clinical Psychology.

[bib3] Amir N., Bomyea J., Beard C. (2010). The effect of single-session interpretation modification on attention bias in socially anxious individuals. Journal of Anxiety Disorders.

[bib4] Amir N., Taylor C.T. (2012). Combining computerized home-based treatments for generalized anxiety disorder: an attention modification program and cognitive behavioral therapy. Behavior Therapy.

[bib5] Beard C., Amir N. (2008). A multi-session interpretation modification program: changes in interpretation and social anxiety symptoms. Behaviour Research and Therapy.

[bib6] Beard C., Weisberg R.B., Amir N. (2011). Combined cognitive bias modification treatment for social anxiety disorder: a pilot trial. Depression and Anxiety.

[bib7] Bishop S.J. (2007). Neurocognitive mechanisms of anxiety: an integrative account. Trends in Cognitive Sciences.

[bib9] Brosan L., Hoppitt L., Shelfer L., Sillence A., Mackintosh B. (2011). Cognitive bias modification for attention and interpretation reduces trait and state anxiety in anxious patients referred to an out-patient service: results from a pilot study. Behavior Therapy and Experimental Psychiatry.

[bib10] Hallion L.S., Ruscio A.M. (2011). A meta-analysis of the effect of cognitive bias modification on anxiety and depression. Psychological Bulletin.

[bib11] Hayes S., Hirsch C.R., Krebs G., Mathews A. (2010). The effects of modifying interpretation bias on worry in generalised anxiety disorder. Behaviour Research and Therapy.

[bib12] Hertel P., Mathews A. (2011). Cognitive bias modification: past perspectives, current findings and future applications. Perspectives on Psychological Science.

[bib13] Hirsch C.R., Hayes S., Mathews A. (2009). Looking on the bright side: accessing benign meanings reduces worry. Journal of Abnormal Psychology.

[bib14] Hoppitt L., Mathews A., Yiend J., Mackintosh B. (2010). Cognitive mechanisms underlying the emotional effects of bias modification. Applied Cognitive Psychology.

[bib15] Mackintosh B., Mathews A., Yiend J., Ridgeway V., Cook E. (2006). Induced biases in emotional interpretation influence stress vulnerability and endure despite changes in context. Behavior Therapy.

[bib33] MacLeod C., Soong L.Y., Rutherford E.M., Campbell L.W. (2007). Internet-delivered assessment and manipulation of anxiety-linked attentional bias: validation of a free-access attentional probe software package. Behavior Research Methods.

[bib17] Mathews A., Mackintosh B. (1998). A cognitive model of selective processing in anxiety. Cognitive Therapy and Research.

[bib18] Mathews A., Mackintosh B. (2000). Induced emotional interpretation bias and anxiety. Journal of Abnormal Psychology.

[bib19] Mathews A., Ridgway V., Cook E., Yiend J. (2007). Inducing a benign interpretational bias reduces trait anxiety. Journal of Behavior Therapy and Experimental Psychiatry.

[bib21] Salemink E., van den Hout M. (2010). Validation of the “recognition task” used in the training of interpretation biases. Journal of Behavior Therapy and Experimental Psychiatry.

[bib22] Salemink E., van den Hout M., Kindt M. (2007). Trained interpretive bias: validity and effects on anxiety. Journal of Behavior Therapy and Experimental Psychiatry.

[bib23] Salemink E., van den Hout M., Kindt M. (2009). Effects of positive interpretive bias modification in highly anxious individuals. Journal of Anxiety Disorders.

[bib24] Schmidt N.B., Richey J.A., Buckner J.D., Timpano K.R. (2009). Attention training for generalized social anxiety disorder. Journal of Abnormal Psychology.

[bib25] Schweizer S., Hampshire A., Dalgleish T. (2011). Extending brain training to the affective domain: increasing cognitive and affective executive control through emotional working memory training. PLoS ONE.

[bib26] See J., MacLeod C., Bridle R. (2009). The reduction of anxiety vulnerability through the modification of attentional bias: a real-world study using a home-based cognitive bias modification procedure. Journal of Abnormal Psychology.

[bib27] Spielberger C.D., Gorsuch R.L., Lushene R., Vagg P.R., Jacobs G.A. (1983). Manual for the state-trait anxiety inventory.

[bib28] Turner R., Hoppitt L., Hodgekin J., Mackintosh B., Fowler D. (2011). Cognitive bias modification in the treatment of social anxiety in early psychosis: a single case series. Behavioural and Cognitive Psychotherapy.

[bib29] Watson D., Clark L.A., Tellegen A. (1988). Development and validation of brief measures of positive and negative affect: the PANAS scales. Journal of Personality and Social Psychology.

[bib30] Watson D., Friend R. (1969). Measurement of social-evaluative anxiety. Journal of Consulting and Clinical Anxiety.

[bib31] Wilson E.J., MacLeod C., Mathews A., Rutherford E.M. (2006). The causal role of interpretative bias in anxiety reactivity. Journal of Abnormal Psychology.

[bib32] Yiend J., Mackintosh B., Mathews A. (2005). Enduring effects of induced interpretation bias. Behaviour Research and Therapy.

